# Positron emission tomography imaging of the sodium iodide symporter senses real-time energy stress in vivo

**DOI:** 10.1186/s40170-023-00314-2

**Published:** 2023-09-07

**Authors:** Piotr Dzien, Agata Mackintosh, Gaurav Malviya, Emma Johnson, Dmitry Soloviev, Gavin Brown, Alejandro Huerta Uribe, Colin Nixon, Scott K. Lyons, Oliver Maddocks, Karen Blyth, David Y. Lewis

**Affiliations:** 1 Cancer Research UK Beatson Institute, Garscube Estate, Switchback Road, Glasgow, G61 1BD UK; 2 School of Cancer Sciences, University of Glasgow, Glasgow, G61 1QH UK; 3 Cold Spring Harbor Laboratory, 1 Bungtown Road, Cold Spring Harbor, NY 11724 USA

**Keywords:** Positron emission tomography, Reporter genes, Metabolic sensor, Energy charge, 2-DG, Oligomycin A, IACS-010759

## Abstract

**Background:**

Tissue environment is critical in determining tumour metabolic vulnerability. However, in vivo drug testing is slow and waiting for tumour growth delay may not be the most appropriate endpoint for metabolic treatments. An in vivo method for measuring energy stress would rapidly determine tumour targeting in a physiologically relevant environment. The sodium-iodide symporter (NIS) is an imaging reporter gene whose protein product co-transports sodium and iodide, and positron emission tomography (PET) radiolabelled anions into the cell. Here, we show that PET imaging of NIS-mediated radiotracer uptake can rapidly visualise tumour energy stress within minutes following in vivo treatment.

**Methods:**

We modified HEK293T human embryonic kidney cells, and A549 and H358 lung cancer cells to express transgenic NIS. Next, we subjected these cells and implanted tumours to drugs known to induce metabolic stress to observe the impact on NIS activity and energy charge. We used [^18^F]tetrafluoroborate positron emission tomography (PET) imaging to non-invasively image NIS activity in vivo.

**Results:**

NIS activity was ablated by treating HEK293T cells in vitro, with the Na^+^/K^+^ ATPase inhibitor digoxin, confirming that radiotracer uptake was dependent on the sodium–potassium concentration gradient. NIS-mediated radiotracer uptake was significantly reduced (− 58.2%) following disruptions to ATP re-synthesis by combined glycolysis and oxidative phosphorylation inhibition in HEK293T cells and by oxidative phosphorylation inhibition (− 16.6%) in A549 cells in vitro. PET signal was significantly decreased (− 56.5%) within 90 min from the onset of treatment with IACS-010759, an oxidative phosphorylation inhibitor, in subcutaneous transgenic A549 tumours in vivo, showing that NIS could rapidly and sensitively detect energy stress non-invasively, before more widespread changes to phosphorylated AMP-activated protein kinase, phosphorylated pyruvate dehydrogenase, and GLUT1 were detectable.

**Conclusions:**

NIS acts as a rapid metabolic sensor for drugs that lead to ATP depletion. PET imaging of NIS could facilitate in vivo testing of treatments targeting energetic pathways, determine drug potency, and expedite metabolic drug development.

**Supplementary Information:**

The online version contains supplementary material available at 10.1186/s40170-023-00314-2.

## Background

Cancer metabolic vulnerabilities depend on complex interactions between tumour genetics, environment and cell-of-origin [1]. Phenotypic differences between tumours and in vitro models, including differential nutrient utilisation, mean that cell-based systems can be unreliable when predicting in vivo drug efficacy [2]. Testing metabolic treatments in vivo, on the other hand, requires lengthy studies that often focus on tumour growth delay and can miss subtle effects on tumour metabolism. What is required is an in vivo system with a rapid readout of drug efficacy downstream of metabolic pathway alterations, which would indicate target engagement and tumour response.

Imaging reporter genes, originally developed to provide an in vivo readout of gene therapy delivery, are now routinely used to investigate a wide range of biological processes in animal models [3]. Thanks to their sensitivity, signal specificity and the relative ease of introduction into tissues of interest, a number of in vivo imaging reporter genes have successfully been used in animal models of cancer to measure tumour treatment response [4, 5]. This has typically been achieved by repeated measurements of the reporter gene signal from expressing tumour cells over the course of treatment with the assumption that the signal is proportional to the number of expressing cells. This is probably adequate when investigating cytotoxic therapies where the signal is produced by viable cells and does not persist in necrotic cells or their remnants. However, this approach does not exploit the possibility of detecting responses to therapeutic interventions that dynamically modulate reporter gene signal, which could provide a real-time readout of the on-target treatment effects independently of whether the treatment subsequently results in cell death.

The enzymatic activity of firefly Luciferase reporter gene (fLuc), for example, depends on the capacity for ATP re-synthesis, one of the hallmarks of cell viability. In the context of assessing cell response to treatment, fLuc’s light output could provide a fast and direct readout of metabolic competence displayed by cells expressing this reporter gene. Although it is conceptually easy to extend this approach to in vivo imaging, complicated kinetics of luciferase signal and low penetration depth inherent to bioluminescence imaging, limit its potential utility [6]. While similar to fLuc in its direct dependence on metabolic competence of the expressing cell, the signal produced by the sodium/iodide symporter (NIS) reporter gene benefits from the high sensitivity and penetration depth afforded by radionuclide imaging. The utility of NIS as a radionuclide reporter gene has been demonstrated in a range of experimental contexts [7–9]. The first use of [^18^F]tetrafluoroborate (TFB) with the NIS reporter gene was followed by the successful imaging human NIS in thyroid cancer patients [10, 11]. Sensitive [^18^F]TFB PET/CT imaging of tumour and metastases has also been demonstrated in vivo via adoptive transfer of NIS-expressing cells [12]. NIS has been adapted using [^18^F]tetrafluoroborate (TFB) (or β-emitting iodide isotopes, such as ^124^I) or [^99m^Tc]TcO_4_^−^ as radioactive probes for PET and SPECT imaging respectively. NIS, encoded by the *Slc5a5* gene (solute carrier family 5 member 5), is an 87 kDa transmembrane glycoprotein with 13 transmembrane domains, which co-imports one iodide anion (I^−^) into the cytosol along with two sodium cations (Na^+^) moving down the Na^+^ gradient established predominantly by the ATP-driven Na^+^/K^+^ ATPase transmembrane exchanger protein [13]. Importantly, in contrast to imaging the HSV1-tk (herpes simplex virus type 1 thymidine kinase) PET reporter gene, whose signal is based on tracer phosphorylation and trapping [3], NIS’ signal is reversible, which allows dynamic measurements of its activity.

Here we show that changes in NIS-mediated radiotracer accumulation induced directly, by pharmacological Na^+^/K^+^ ATPase inhibition, or indirectly, by drugs inhibiting ATP re-synthesis, can be detected minutes from the onset of treatment. Using measurements of radiotracer uptake in vitro and sensitive PET imaging in vivo, we obtained a rapid, on-target readout of tumour response to agents modulating cancer cell metabolism.

## Methods

### Materials

Cell culture media: DMEM (#11,965,092), RPMI1640 (#21,870,084) and L-Glutamine (#25,030,081) were purchased from ThermoFisher Scientific (Life Technologies). Oligomycin A, 2-deoxyglucose, Digoxin, Matrigel®, methylcellulose, DMSO, analytical grade acetonitrile and methanol were purchased from Merck Life Science UK (Sigma Aldrich). IACS-10759 [14, 15] was purchased from Chemietek (Indianapolis, IN, US).

### Radiopharmaceuticals

Saline solution of [^99m^Tc]Pertechnetate for intravenous injection was purchased from the West of Scotland Radionuclide Dispensary, NHS Greater Glasgow and Clyde, and diluted in cell culture media before use. [^18^F]Tetrafluoroborate sodium salt was produced at the radiopharmaceutical unit of the West of Scotland Glasgow PET Centre by the in-house developed radiolabeling procedure [16]. This afforded [^18^F]TFB radiopharmaceutical preparation in sterile saline with radiochemical purity exceeding 98%, and molar activity in the range of 20–100 GBq/μmol at the time of injection. The solution was diluted, as needed, in saline before use.

### Cell culture

NIH A549, H358, and HEK293T cell lines were purchased from ATCC. Absence of mycoplasma was confirmed by regular in-house testing. HEK293T cells were grown in DMEM and A549 and H358 cells in RPMI1640, supplemented with 2 mM Glutamine and 10% FBS (Gibco™). Drugs for in vitro use were prepared as follows: Digoxin and oligomycin A: 10 mM stock was prepared in DMSO, diluted in DMEM to 100 µM and used at 1:100; 2-DG: 300 mM stock was prepared in distilled H_2_O and used at 1:100; IACS-10759: 10 mM stock was prepared in DMSO, diluted in complete RPMI to 10 µM, and used at 1:1000, 1:200, or 1:100.

### Generation of mNIS-expressing clonal cell lines

Lentiviral vectors carrying either Strawberry-P2A-mNIS or Luc2-P2A-mNIS (referred to as “SN” and “LN”, respectively) reporter gene cassette under the *PGK* promoter [17] were produced essentially as described previously [18]. Briefly, 80% confluent HEK293T cells were transfected with the three packaging plasmids and the SN or the LN transfer plasmids using the Lipofectamine protocol (Life Technologies, Thermo Fisher Scientific). At 48 h after transfection, virus-containing supernatants were removed, centrifuged for 5 min at 800 g at 4 °C and passed through a syringe-driven 45 µm low-protein-binding filter.

For transduction, target cells were seeded in 6-well plates and infected with 1.0–1.5 mL of the lentivirus-containing supernatant per well 16–24 h later. HEK293T cells were transduced with the SN and A549 and H358 cells with the LN lentiviral vectors, respectively. Infected cells were harvested by trypsinisation 72 h post-infection, seeded singly in 96-well plates and grown to confluence. Transgene expression in the resulting colonies was confirmed by fluorescence microscopy (Strawberry) or bioluminescence imaging (Luc2). Strawberry-positive (HEK293T-SN), Luc2-positive (A549-LN), and (H358-LN) clonal cell colonies were expanded and mNIS expression in these was confirmed by [^18^F]TFB uptake experiments.

### Metabolic characterisation of mNIS-expressing cells and their response to treatment

Cells were seeded, typically at 2 × 10^5^ cells per well, in 6-well plates in normal growth medium and incubated in 5% CO_2_ at 37 °C for 48–72 h. For assay experiments, the medium was removed and fresh growth medium, supplemented with the drugs, or their vehicles and, in the case of uptake assays, radioactive tracers, were added to the cultures in 1 mL total volume. Protein concentration was assayed using the Pierce™ BCA (bicinchoninic acid) Protein Assay Kit (ThermoFisher Scientific). Cell counting and trypan blue exclusion assays were performed using CellDrop Automated Cell Counters (DeNovix). Experiments testing each of the conditions, including cell counts for LC–MS extractions (see below), used three biological replicates, unless stated otherwise. Individual data points were normalised to the mean of control for statistical analysis and presentation. For radioactivity measurements, decay-corrected counts were normalised to cell numbers counted in plates cultured in parallel, which had been treated as per the tested conditions, except that no radioactivity was added.

Ordinary, one-way, multiple comparisons ANOVA was performed to test statistical significance of the results, and *P* values calculated using Dunnett’s multiple comparisons test, comparing every condition against the control, were reported.

### Cell extraction for LC–MS analysis

Briefly, 100 µL samples of growth medium were withdrawn from each well 5 min before the end of incubation and centrifuged for 10 min at 16,200 g and 4 °C to remove debris. Next, 10 µL samples of the supernatant were then extracted in 990 µL of ice-cold Methanol–Acetonitrile-Water (50%:30%:20%) extraction buffer and kept at 4 °C. Shortly before the extraction the number of cells was estimated from counts that were performed in replicate plates, seeded and grown in parallel to those used in the extractions, and volume of the extraction buffer was adjusted to 0.4 mL per 10^6^ cells. At the end of incubations (at 30, 60, or 120 min from the addition of conditioned medium) the medium was removed rapidly, cells were washed with 5–10 mL of ice-cold PBS, and metabolites extracted by the addition of ice-cold extraction buffer, followed by 10 min incubation on ice and 15 min gentle rocking at 4 °C. Cell extracts were transferred to 1.5 mL Eppendorf tubes and centrifuged for 10 min at 16,200 g and 4 °C, using Eppendorf 5415D benchtop centrifuge to remove debris. Samples containing 0.2 mL of the cell or media extracts were transferred to liquid chromatography vials and stored at  -80 °C.

### Liquid chromatography–high-resolution mass spectrometry (LC–HRMS)

LC-HRMS was performed in an Accela 600 LC system (Thermo Fisher Scientific) coupled to an Exactive (Orbitrap) mass spectrometer (Thermo Fisher Scientific). Metabolite separation was done using a SeQuant ZIC-pHILIC column (4.6 mm × 150 mm, 5 μm) (Merck). The mobile phase consisted of an aqueous solution of ammonium carbonate (20 mM, pH 9.2) (A) and acetonitrile (B) and the following gradient profile was applied: linear increase of A from 20 to 80% at 0 to 30 min, 92% A 31–37 min and linear decrease of A to 20% at 37–46 min. Total injection volume was 20 µL and samples were maintained at 4 °C throughout analysis. The spectrometer was operated in both positive and negative electrospray ionisation (ESI) modes, full-scan mode over a mass range of m/z 70–1200 at a resolution of 50,000. The capillary temperature was 320 °C and the seath and auxiliary gas flow rates were 50 and 17 units, respectively. Thermo raw files were converted to mzML files using ProteoWizard, separated into ESI positive and negative modes, and imported to MZMine 2.53 for peak processing. Metabolite identification was performed using an in-house standard library and peak areas were exported for analysis. The (peak area) energy charge ratio (ECR) was calculated from the formula ECR = ([ATP] + 0.5 [ADP])/([ATP] + [ADP] + [AMP]), where [metabolite] is the metabolite’s peak area [19].

### In vitro radioactivity uptake experiments

Samples (1 mL) of growth medium with either drugs or their vehicles and containing, at application time, approximately 50 kBq/mL of [^99m^Tc]pertechnetate or [^18^F]TFB, were added per well to 6-well plates containing tested cells. At the end of incubations, between 30 and 120 min from the addition of radioactivity, the media were removed rapidly and the plates were washed immediately with 5–10 mL of ice-cold PBS per well. Cells were lysed by the addition of 1 mL of RIPA lysis and extraction buffer (radioimmunoprecipitation assay, ThermoFisher Scientific, Life Technologies) per well, 10 min incubation on ice and 15 min gentle rocking at 4 °C. Radioactivity of the lysates was measured using Hidex Automatic Gamma Counter (Hidex Oy, Turku, Finland). Events within the energy window of 15–200 keV ([^99m^Tc]pertechnetate) or 400–800 keV ([^18^F]TFB) were used in analysis.

### Tumour cell implantation and treatment

Nine to 14-week-old NOD/NcrCrl (*Prkdc*^scid^) mice were purchased from Charles River UK. Shortly before implantation, A549-LN and H358-LN cells were harvested by trypsinisation and re-suspended in an ice-cold, 50:50 mixture of serum-free RPMI and Matrigel® (Corning). To establish xenografts, 100 µL samples of cell suspensions, each containing 5 × 10^6^ cells, were injected subcutaneously in the right flank. Xenograft diameters were measured with callipers, and the length (L) and width (W) were used to calculate the volume (V) using the simplified ellipsoid formula:

$${\varvec{V}}=\frac{1}{6} \pi L{W}^{2}$$

IACS-10759 as a 2 mg/mL suspension in vehicle (0.2% solution of methylcellulose in distilled water), or vehicle alone, were administered by oral gavage at 10 µL per g body weight.

### PET-MR imaging and data analysis

Animals were imaged approximately 4 weeks from cell implantation. Immediately after IACS-10759 or vehicle administration animals were anaesthetised with 1.0–2.5% isoflurane in 95% oxygen and a cannula was inserted into the tail vein. At 15–20 min from IACS-10759 or vehicle administration animals were injected intravenously with 0.35–0.45 MBq of [^18^F]TFB per g body weight in 200–250 µL saline (0.9% NaCl) and transferred to a NanoScan PET/MRI (1T) (Mediso, Hungary). Respiration rate of the animals was monitored by pneumatic pad for the duration of the imaging session and their body temperature was maintained by flow of heated air. Coronal T1-weighted images, used for anatomical reference and attenuation correction, were acquired using 3D gradient-recalled echo sequence (TR 22.5 ms; TE 3.8 ms; flip angle 30°; data matrix, 256 × 256; slice thickness 0.70 mm; 48 slices). A 20 min static PET image was then acquired, starting 70 min from the injection of [^18^F]TFB.

Image reconstruction was performed using 3D Tera-Tomo software (Mediso Medical Imaging Systems, Hungary). PET scans were reconstructed using static, total-body mode with 4 iterations and 6 subsets and an energy window 400–600 keV, producing a 0.4 mm isotropic matrix. PET data were corrected for radioactivity decay, random coincidences, scatter, attenuation, and dead time. Scatter and attenuation correction used the T1 3D GRE MR images. The reconstructed PET scans were co-registered with MRI scans for anatomical reference. PET/MR data were analysed using VivoQuantTM multi-modality post-processing suite (Invicro, USA). SUVmax was calculated with regions of interest over the whole of the tumour volume.

Maximum standardized uptake values (SUV) were calculated using.

$${\text{SUV}}=\frac{\mathrm{c\,img}}{ID/BW}$$

where c_img_ is the activity concentration (MBq/mL) derived from the image ROI, ID is the injected dose, and BW is the body weight of the animal.

### Long-term treatment with IACS-10759

Starting at day 23 after the tumour cell implantation the cohorts was randomly divided into two groups treated daily for 6 consecutive days with IACS-10759 (the test group; *n* = 4) or vehicle (the control group; *n* = 4). Calliper measurements of the tumours were taken at 24 h before the first dose (‘0 h’), 24 and 96 h after the first dose (’48 h’ and ‘120 h’, respectively), and 24 h after the sixth dose (‘168 h’). Shortly after the last calliper measurement the animals were sacrificed by cervical dislocation, the tumours rapidly excised and fixed in 4% NBF.

### Immunohistochemistry (IHC) staining

All IHC staining was performed on 4 µm formalin fixed paraffin embedded sections (FFPE) which had been baked in the oven at 60 °C for 2 h.

FFPE sections for pAMPKα (2535, Cell Signaling) and pPDHA1 (ab177461, Abcam) staining were loaded into an Agilent pre-treatment module for dewaxing and heat induced epitope retrieval (HIER) using high pH target retrieval solution (TRS) (K8004, Agilent). Sections were heated to 97 °C for 20 min in high TRS buffer. Sections were then rinsed in flex wash buffer (K8007, Agilent) and loaded onto the Agilent autostainer link48. The sections underwent peroxidase blocking (S2023, Agilent) for 5 min and were rinsed with flex wash buffer before applying primary antibody to the sections at a previously optimised dilution (pAMPKα, 1:75; pPDHA1, 1:100) for 35 min. The sections were washed with flex wash buffer before application of rabbit envision secondary antibody (K4003, Agilent) for 30 min. Sections were rinsed with flex wash buffer before applying Liquid DAB (K3468, Agilent) for 10 min. The sections were washed in water and counterstained with Haematoxylin Z (RBA-4201-00A, CellPath).

FFPE sections for Caspase 3 (9661, Cell Signaling), Glut-1 (GT12-A, Alpha Diagnostics) and Ki67 (12202, Cell Signaling) investigation were stained on a Leica Bond Rx autostainer undergoing on-board dewaxing (AR9222, Leica) and antigen retrieval using ER2 solution (AR9640, Leica) for 20 min at 95 °C. Sections were rinsed with Leica wash buffer (AR9590, Leica) before peroxidase block was performed using an Intense R kit (DS9263, Leica). FFPE sections were rinsed with wash buffer and primary antibodies applied at a previously optimised dilution (Caspase 3, 1:500; Glut-1, 1:75; Ki67, 1:1000). The sections were rinsed with wash buffer and rabbit envision secondary antibody applied for 30 min. The sections were rinsed with wash buffer, visualised using DAB and then counterstained with Haematoxylin in the Intense R kit. To complete the IHC staining sections were rinsed in tap water, dehydrated through graded ethanol’s and placed in xylene. The stained sections were coverslipped in xylene using DPX mountant (CellPath, UK).

### IHC image analysis

Slides were scanned at ×20 magnification using Leica Aperio AT2 instrument and analysed using HALO image analysis platform (Indica Labs, Albuquerque, NM, USA). HALO CytoNuclear v1.6 macro, tuned to identify negative (N) and weakly (W), moderately (M) or strongly (S) DAB-stained cells. H-score, defined below, was reported:

$${\varvec{H}}-{\varvec{S}}{\varvec{c}}{\varvec{o}}{\varvec{r}}{\varvec{e}}=\frac{3\mathrm{S }+2\mathrm{M}+1\mathrm{W}}{\mathrm{S}+\mathrm{M}+\mathrm{W}+\mathrm{N }}\times 100$$

### Statistical analysis

Prism 9.0 (GraphPad) was used to perform statistical analysis and to plot the data. In figures, error bars represent one standard deviation. *P* value classifications are summarized as follows: ns, *P* > 0.05; *, P ∈ (0.01–0.05 〉; **, *P* ∈ (0.001–0.01〉; ***, *P* < 0.001.

## Results

### NIS signal in HEK293T-SN cells is decreased in response to drugs targeting Na^+^/K^+^ ATPase, glycolysis and oxidative phosphorylation

**Fig. 1:**
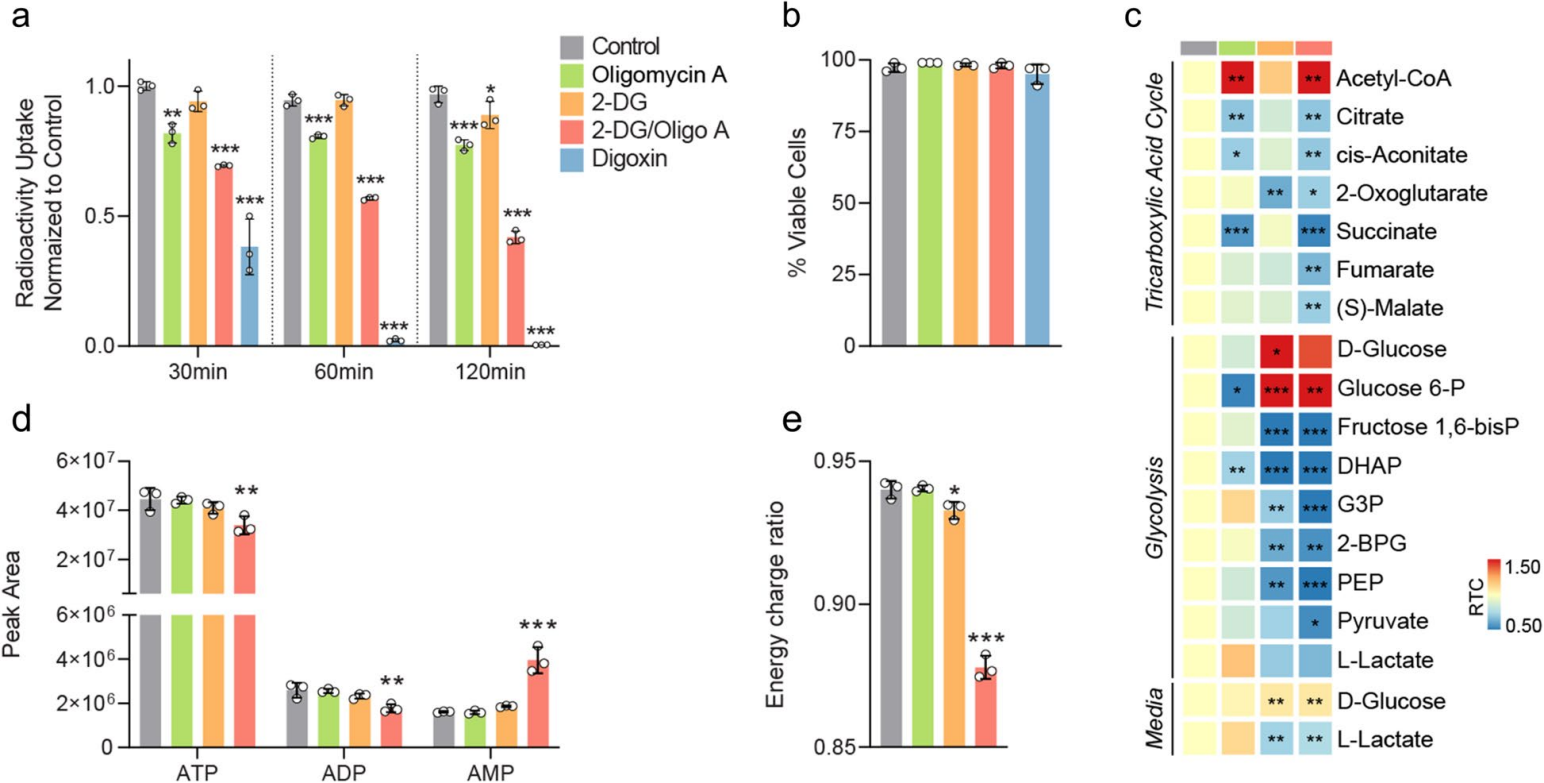
NIS signal in HEK293T-SN cells is reduced in response to drugs targeting NaK ATPase, glycolysis and oxidative phosphorylation. **a** Radioactivity uptake in HEK293T-SN cells in vitro at 30, 60, and 120 min, respectively, after the addition of [^18^F]TFB at 50 kBq/mL and drugs at specified concentrations. Decay-corrected radioactivity counts were normalised to cell numbers estimated using replicate plates, conditioned for 120 min as the cell cultures used in radioactivity uptake assay, but without added radioactivity. For presentation, individual data points were normalised to the mean of control. **b** Results of trypan blue exclusion assay performed at 120 min from the beginning of conditioning as specified in **a**. **c** LC-MS measurements of selected intra- and extracellular metabolites at 120 min from the beginning of drug conditioning. Volume of extraction buffer was adjusted to 1 mL per 2.5 × 10^6^ cells according to cell counts obtained from replicate plates, conditioned for 120 min as per plates used in the extraction. **d** LC-MS measurements of cell adenine nucleotides collected in experiments described in **c**. **e **Energy charge ratio (ECR) calculated for LC-MS experiments described in **c** and **d** from the formula ECR = ([ATP] + 0.5 [ADP])/([ATP] + [ADP] + [AMP])**, **where [metabolite]is the metabolite’s peak area. Ordinary, one-way, multiple comparisons ANOVA was performed to test statistical significance of the results, and *P* values calculated using Dunnett’s multiple comparisons test, evaluating all conditions versus the control at each respective time point were presented. *P* value classifications are summarized as follows: *, *P*∈(0.01–0.05 〉; **, *P*∈(0.001–0.01 〉;  ***, *P* < 0.001. Only statistically significant (*P* < 0.05) results are presented. All experiments used *n* = 3 biological replicates, each represented by a data point. Error bars represent one standard deviation.

We hypothesised that Na^+^/K^+^ ATPase inhibition [9, 13], either directly by digoxin or indirectly via inhibition of ATP re-synthesis pathways such as oxidative phosphorylation, glycolysis or their combination, would lead to rapid dissipation of the Na^+^ gradient and result in a decrease in NIS activity, measured by reduced radiotracer uptake. To test this, we treated HEK293T-SN cells, expressing transgenic NIS, with the Na^+^/K^+^ ATPase inhibitor, digoxin, in vitro. This resulted in a collapse of NIS signal as early as 30 min from the onset of treatment (Fig. 1a), an effect which cannot be explained by gross changes in plasma membrane integrity, which appeared unaffected (Fig. 1b), demonstrating the dependency of NIS on functioning Na^+^/K^+^ ATPase.

To determine if NIS was also dependent on treatment targeting energetic pathways we treated HEK293T-SN cells with oligomycin A, a mitochondrial ATP synthase inhibitor [20], which resulted in pronounced changes in tricarboxylic acid cycle (TCA) intermediate levels, including decreases in citrate, cis-aconitate and succinate, and an increase in acetyl-CoA (Fig. 1c), consistent with inhibition of oxidative phosphorylation. This led to a compensatory increase in glycolytic flux, as indicated by a decrease in intracellular glucose 6-phosphate and a trend towards increased intra- and extracellular lactate concentrations (Fig. 1c). While we measured a modest decrease in NIS-mediated radioactivity uptake, there was no indication that disruption of ATP re-synthesis was responsible for this, as intracellular adenine nucleotide (ATP, ADP, and AMP) levels appeared unaffected (Fig. 1d). The decrease in NIS activity observed here can be accounted for by the previously reported off-target effects of oligomycin A, which is known to directly inhibit Na^+^/K^+^ ATPase [21, 22], albeit significantly less so than digoxin.

Administration of glucose metabolism antagonist, 2-deoxyglucose (2DG) [23], inhibited glycolytic flux, as evidenced by the decrease of glucose uptake from the medium and decreased lactate excretion (Fig. 1c). While only small, statistically insignificant decreases in ATP and ADP, and a small increase in AMP levels were detected (Fig. 1d), the modest reduction of the energy charge ratio (Fig. 1e) points to disruption of ATP re-synthesis as the event linking 2DG administration with NIS inhibition.

Treatment combining oligomycin A and 2DG produced metabolic hallmarks of oxidative phosphorylation and glycolysis inhibition, respectively—decreased citrate, cis-aconitate and succinate levels or decreased glucose uptake and lactate output (Fig. 1c). Simultaneous inhibition prevented compensation between these two key ATP re-synthesis pathways and resulted in a decrease in ATP concentration and a sharp increase in AMP concentration (Fig. 1d), reflected in the decreased energy charge ratio (Fig. 1e). Consequently, a pronounced and progressive decrease in NIS activity was detectable as early as 30 min from the onset of treatment (Fig. 1a), linking energy stress to NIS activity in HEK293T-SN cells.

### NIS signal in A549-LN cells is dose-dependently decreased following oxidative phosphorylation inhibition

**Fig. 2:**
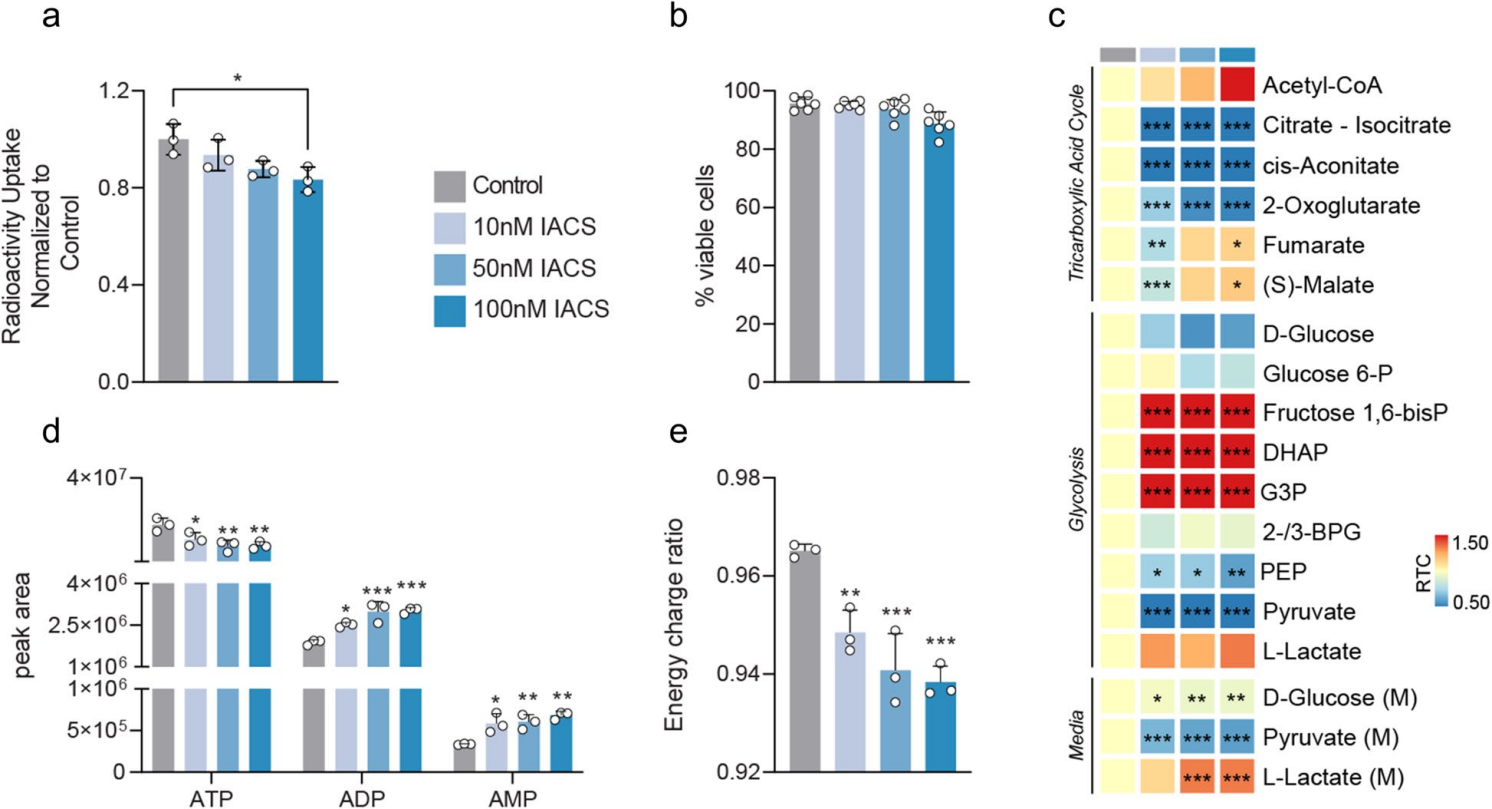
NIS signal in A549-LN cells is dose-dependently decreased following oxidative phosphorylation inhibition. **a** Radioactivity uptake in A549-LN cells in vitro at 60 min from the addition of ^99m^Tc at 50 kBq/mL and vehicle or IACS-10759, at specified concentrations. Decay-corrected radioactivity counts were normalised to protein concentrations assayed in samples of lysates used in radioactivity measurements. For presentation, individual data points were normalised to the mean of control. **b** Summarised results of trypan blue exclusion assay performed in separate set of cell cultures at 60 min from the beginning of drug conditioning as described in **a**. **c** LC-MS measurements of selected intra- and extracellular metabolites extracted at 60 min from the beginning of IACS-10759 conditioning. Volume of the extraction buffer was adjusted to 1 mL per 2.5 × 10^6^ cells according to cell counts obtained from replicate plates, conditioned as per plates used in the extraction. **d** LC-MS measurements of adenine nucleotide concentrations collected in experiments described in **c**. **e** Energy charge ratio (ECR) calculated for LC-MS experiments described in **c** and **d** from the formula ECR = ([ATP] + 0.5 [ADP])/([ATP] + [ADP] + [AMP])**,** where [metabolite] is the metabolite’s peak area. Ordinary, one-way, multiple comparisons ANOVA was performed to test statistical significance of the results, and *P* values calculated using Dunnett’s multiple comparisons test, evaluating all conditions versus the control at each respective time point were presented. *P* value classifications are summarized as follows: *, *P* ∈ (0.01–0.05 〉; **, *P* ∈ (0.001–0.01 〉; ***, *P* < 0.001. Only statistically significant (*P* < 0.05) results are presented. All experiments used *n* = 3 or *n* = 6 (for trypan blue assay) biological replicates, each represented by a data point. Error bars represent one standard deviation

To determine the utility of NIS to measure response in a therapeutically relevant context we treated lung A549-LN cancer cells expressing transgenic NIS with the electron transport chain complex I inhibitor, IACS-10759 [14, 15]. A549-LN cells were derived from the A549 human non-small cell lung cancer line which has increased sensitivity to oxidative phosphorylation inhibition due to a mutation of the SWI/SNF chromatin remodelling complex member, SMARCA-4 [14]. Oxidative phosphorylation inhibition by IACS-10759 led to a pronounced decrease in the concentrations of TCA intermediates citrate, cis-aconitate and 2-oxoglutarate, and triggered a compensatory increase of glycolytic flux, as evidenced by the depletion of extracellular pyruvate and, to a smaller extent, glucose, and by the increase in lactate excretion (Fig. 2c). IACS-10759 administration resulted in a dose-dependent decrease in NIS-mediated radioactivity uptake 60 min after drug administration, independently of cell viability, most likely a result of acute ATP re-synthesis disruption (Fig. 2a, b). Consistent with this, LC–MS measurements indicated decreases in ATP concentration, increases in ADP and AMP concentrations (Fig. 2d) and a decrease in the energy charge ratio (Fig. 2e), again linking energy stress resulting from electron transport chain complex I inhibition to reduced NIS-mediated radiotracer uptake.

### NIS can non-invasively image oxidative phosphorylation inhibition in vivo using PET

We went on to determine if oxidative phosphorylation inhibition by IACS-10759 could be detected rapidly and non-invasively by [^18^F]TFB PET imaging of NIS expressing cells in vivo. To this end, we implanted A549-LN cells in the right flank of NOD/NcrCrl (Prkdc^scid^) mice, which produced tumours reaching volumes of approximately 700 mm^3^ 4 weeks later.

**Fig. 3:**
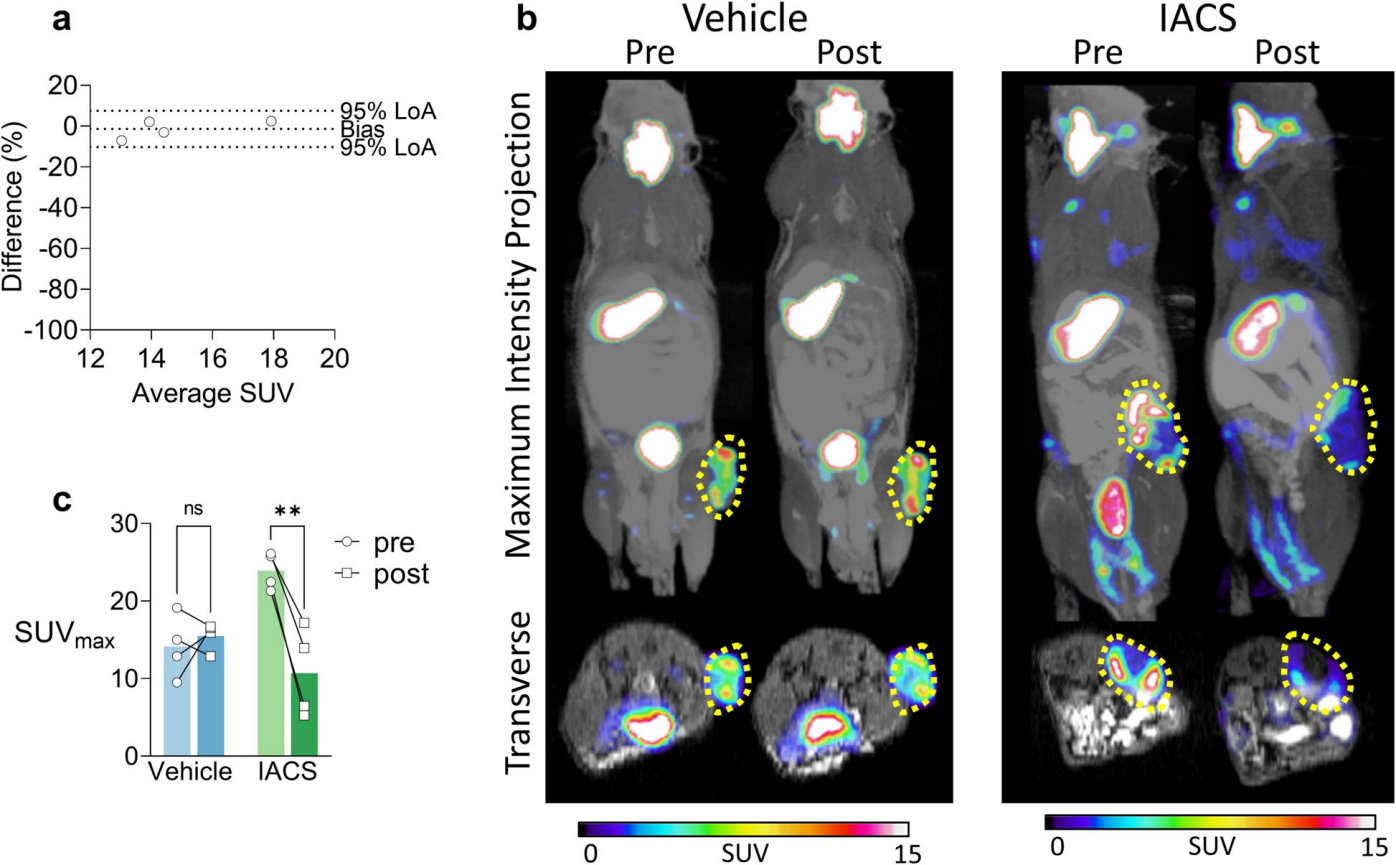
NIS can non-invasively image oxidative phosphorylation inhibition in vivo using PET. **a** Bland-Altman analysis of tumour [^18^F]TFB-PET imaging quantification performed in a cohort of mice (*n* = 4) at baseline and 24 h later, immediately after oral administration of vehicle. Bias and 95% lines of agreement (LoA) are indicated. **b** Example [^18^F]TFB-PET MRI imaging performed 24 h before (‘pre’) and immediately after (‘post’) the administration of the vehicle (left) or IACS-10759 (right). Static PET images were acquired between 70 and 90 min from the injection of [^18^F]TFB and maximum standardised uptake values (SUV_max_) from the region of interest (ROI) placed over the tumour were calculated using VivoQuant image analysis suite (indicated by dashed yellow line). **c** Summary of experiments performed in cohorts (each *n* = 4) treated with a single dose of vehicle (left) or IACS-10759 (right), as described in **a**. Two-way, repeated measures ANOVA with Šídák's multiple comparisons analysis was performed to test statistical significance of the results. *P* value classifications are summarized as follows: *, *P* ∈ (0.01–0.05 〉; **, *P* ∈ (0.001–0.01 〉. Each data point represents SUV_max_ calculated for a single tumour/animal and time-point. Error bars represent one standard deviation

To determine the consistency of [^18^F]TFB PET imaging of NIS activity in the A549-LN model we imaged a cohort of tumour-bearing mice (*n* = 4) twice: once to establish baseline and then 24 h later, immediately after oral administration of the vehicle. A Bland–Altman analysis showed that repeated PET imaging was highly consistent showing minimum bias (-1.4 ± 4.5%) between baseline and day 1 PET imaging, the 95% lines of agreement were between 7.5% and -10.26%, suggesting high precision of PET imaging and good ability to discriminate small changes in NIS activity (Fig. 3a).

Baseline PET uptake was characterised by focal uptake in the tumour region and low background in surrounding tissues (Fig. 3b). We observed the expected physiological uptake of [^18^F]TFB in the thyroid and stomach with excretion through the bladder. [^18^F]TFB PET signal in the tumour was heterogeneous, with punctate signal in some areas and lower uptake close to tumour centre (Fig. 3b). Tumour edge intensity likely reflects typical perfusion pattern in subcutaneous implantation models [24, 25].

To determine the effect of IACS-10759 on NIS activity, a separate cohort of tumour-bearing mice (*n* = 4) received 20 mg/kg IACS-10759 by oral gavage 24 h after baseline imaging and immediately before [^18^F]TFB PET-MR imaging. IACS-10759 administration resulted in a large and consistent decrease (-56.5% ± 20.1%) of [^18^F]TFB PET uptake, which was not observed in mice treated with vehicle (Fig. 3b, c), indicating a specific effect IACS-10759 on NIS activity. Despite the requirement for drug absorption, biodistribution and target engagement this large decrease was detectable within approximately 90 min from oral administration of the drug (including 70 min uptake time for [^18^F]TFB), thus providing a near real-time readout of drug activity. Notably, IACS-10759-induced inhibition of NIS in A549-LN–derived xenografts (Fig. 3c) was significantly more pronounced than for A549-LN cells in vitro (Fig. 2a), which may be a metabolic effect of the different environments to which these cells were exposed, specifically their respective concentrations of extracellular glucose and lactate. Glycolytic substrate-level phosphorylation can compensate for an acute ATP depletion downstream of complex I inhibition, but has an approximately 15-fold lower net yield of ATP per molecule of glucose [26], thus placing a greater demand on glucose uptake rate and the excretion rate of the end product, lactate [27]. It is conceivable, therefore, that in the in vivo scenario described here the rates of glucose delivery and lactate excretion cannot support glycolytic flux at a level sufficient to compensate for oxidative phosphorylation inhibition to the degree observed in vitro.

Finally, we investigated whether [^18^F]TFB PET imaging of NIS activity could differentiate responding and non-responding tumours. To do this, we measured [^18^F]TFB uptake in H358-LN-derived xenografts after inhibiting NIS with IACS-10759 (Figure S2). H358 tumours are resistant to IACS-10759 due to the absence of the SMARCA-4 mutation found in A549 [14]. In contrast to the significant decrease in [^18^F]TFB PET imaging signal in A549-LN, the response in H358-LN was not statistically significant (+ 15.5% ± 11.8%; *P* = 0.12). These findings suggest that utilizing [^18^F]TFB to measure NIS activity can be helpful in distinguishing the response to oxidative phosphorylation inhibition induced by IACS-10759.

### Effects of IACS-10759 on tumour growth and protein markers

**Fig. 4:**
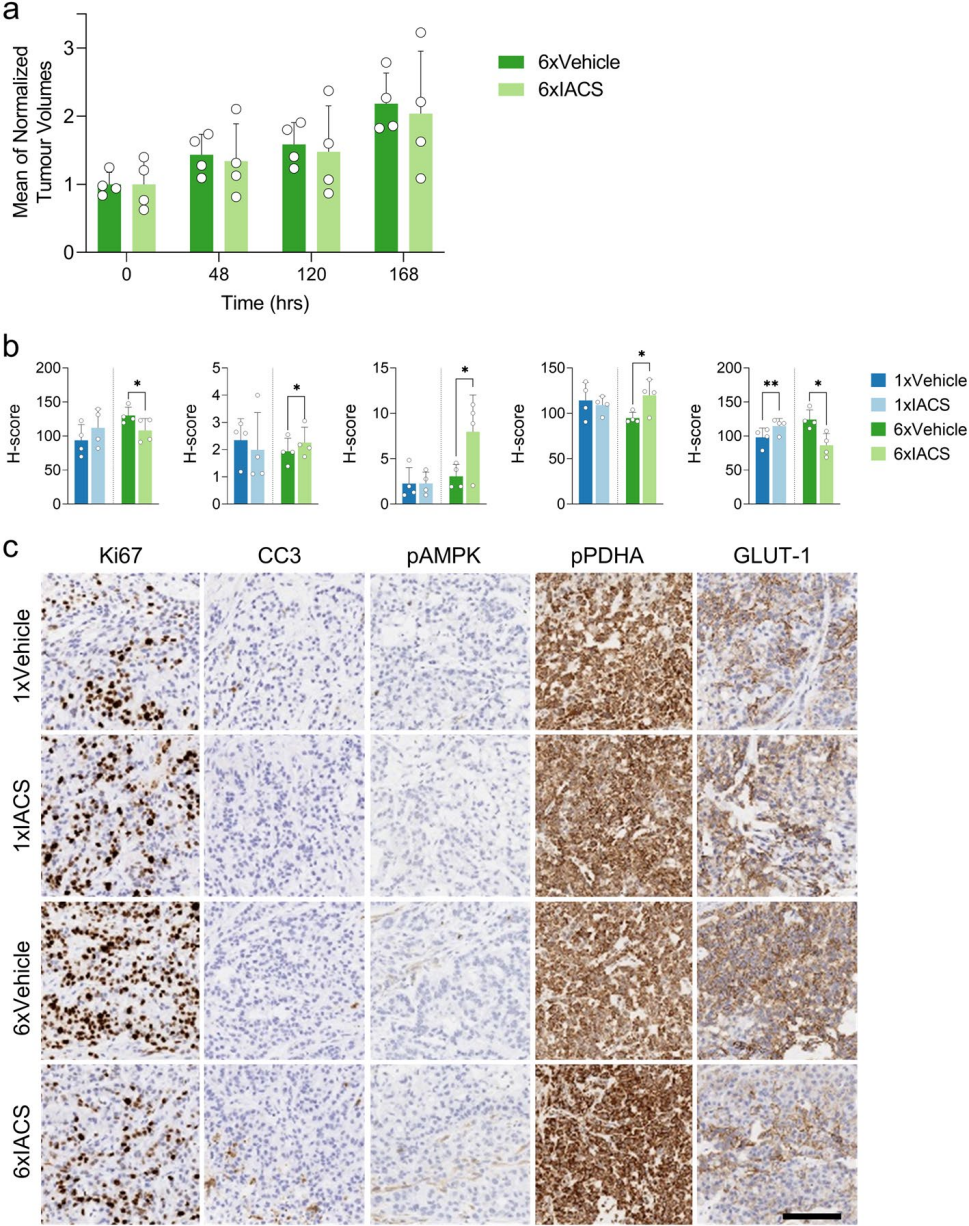
Effects of IACS-10759 treatment on tumour growth and protein markers. **a** Tumour growth time-course under chronic IACS-10759 treatment. Calliper measurements taken at *t* = 0, 48, 120, and 168 h were converted to volumes (*n* = 4 mice per group). Six consecutive, once daily doses of IACS-10759, or vehicle were administered, starting at 24 h after the first calliper measurement. For presentation, individual data points were normalised to the mean of the respective treatment group at *t* = 0 h. **b** Summarised results of IHC staining against (from left to right): Ki67, cleaved caspase 3, phospho-AMPK (Thr172), phospho-PDHA1 (S293) and GLUT1 performed in the respective treatment groups: Vehiclex1, IACSx1, Vehiclex6, IACSx6 (single or six, once-daily doses of vehicle or IACS-10759, respectively), quantified using HALO image analysis platform. One-tailed Student’s *t* test, performed separately for each of the two vehicle—IACS treatment durations (single dose and 6, once-daily doses, respectively) was used to test statistical significance of the results. *P* value classifications are summarized as follows: *, *P* ∈ (0.01–0.05 〉; **, *P* ∈ (0.001–0.01〉. Only statistically significant (*P* < 0.05) results are indicated on the graph. Each data point represents an H-score obtained from an image of a single stained section (*n* = 4 each for both drug treatment durations and their respective vehicle control groups). Error bars represent one standard deviation. **c** Example IHC images showing staining summarised in **b**, with (from left to right): Ki67, cleaved caspase 3, phospho-AMPK (Thr172), phospho-PDHA1 (S293) and GLUT1 and (from top to bottom): Vehiclex1, IACSx1, Vehiclex6, IACSx6. Scale bar indicates 100 μm

To determine the wider effects of IACS-10759 we treated A549-LN xenografts (*n* = 4 each for both treatment durations and respective vehicle controls) with either a single dose or six consecutive once-daily doses of IACS-10759 over 2 or 168 h, respectively, and measured tumour growth and the expression of metabolic enzymes and transporters. Although this relatively short course of treatment did not induce an appreciable decrease in tumour growth rate (Fig. 4a), the expression of phosphorylated (Thr-172) AMPK (AMP-activated protein kinase) and phosphorylated (S-293) PDH (pyruvate dehydrogenase) subunit A1 both increased in treated tumours (Fig. 4b, c), indicating activation of the AMPK metabolic stress sensor and downregulation of TCA cycle flux by PDH complex inhibition, respectively [28]. Increased ATP turnover to ADP stimulates the activity of AK (adenylate kinase) [29], an enzyme converting two ADP molecules to one molecule each of ATP and AMP. While this re-generates ATP, the AMP produced is a signal of energy stress, propagated further via AMPK Thr-172 phosphorylation [30].

Xenograft GLUT1 glucose transporter expression increased shortly after the administration of a single dose of IACS-10759 in vivo (Fig. 4b, c), suggesting an acute upregulation of glucose uptake in response to treatment, similar to what we observed at the metabolite level in vitro, and by implication also indicating acute depletion of intracellular glucose resulting from increased glycolytic flux. GLUT1 is responsible for basal glucose transport, but its expression at the protein level can be upregulated within tens of minutes from the onset of metabolic stress [31, 32]. Decreased expression of GLUT1 was observed after chronic IACS-10759 administration (Fig. 4b, c), which may indicate a switch to alternative carbon sources, such as circulating fatty acids, to maintain ATP re-synthesis. Alternatively, the profile of transmembrane glucose transport proteins may have moved toward other members of the facilitative GLUT transporter family, such as the high-affinity GLUT3, or to sodium gradient-dependent SGLT transporters (human Sodium Glucose Transporters) [31, 33], to maintain a higher rate of glycolytic flux upregulated in response to IACS-10759 administration. Together, these changes suggest a compensatory re-wiring of tumour metabolism in response to metabolic stress induced by inhibition of oxidative phosphorylation. Increased cleaved caspase 3 apoptosis marker staining and decreased staining with the proliferation marker Ki67 in chronically treated tumours indicated that, at least for a relatively small subset of tumour cells, the treatment had a modest cytotoxic and/or cytostatic effect (Fig. 4b, c).

## Discussion

We demonstrate here that measurements of NIS activity can provide a sensitive, real-time readout of energy stress induced by metabolic drugs. The approach using NIS as a metabolic sensor of ATP re-synthesis disruption has a number of advantages. Firstly, it provides a rapid (within minutes) in vivo readout of response to metabolic drugs. This is important, as the efficacy of metabolic treatments is context-dependent, which explains why in vitro drug responses are not always predictive of in vivo efficacy, yet screening metabolic compounds in vivo is slow and inefficient. The proposed approach makes in vivo testing more practical as it can offer an immediate readout after a single dose, reducing drug costs, animal use, attrition, and the time associated with long therapeutic studies. Further, this approach offers a more refined view of metabolic response than volumetric assessment, as it can detect sub-clinical metabolic stress that may not result in growth delay, but may be efficacious when combined with a complementary metabolic therapy to achieve tumour kill.

Secondly, by detecting metabolic stress rather than upstream target engagement, NIS provides a more generic detection method than imaging agents that primarily inform on the flux through a single metabolic node, for example transmembrane uptake and/or phosphorylation of the tracer. Metabolic PET imaging workhorse, [^18^F]FDG (2-Deoxy-2-[^18^F]fluoroglucose) would likely produce a false negative result here, as FDG signal is primarily a readout of glucose uptake and phosphorylation, both of which would increase to support acutely upregulated glycolytic flux. Preclinically, at least, measuring NIS activity as a surrogate of energy stress may also be more useful than imaging agents that target downstream pathways like cell death, which may not be an appropriate endpoint for metabolic treatments.

Thirdly, the scale of the response here (-56.5%) is substantial compared with tracers targeting endogenous pathways, for example measuring changes in mitochondrial membrane potential with [^18^F]fluoroBnTP PET after oxidative phosphorylation inhibition (-20%) [34], changes in oxygen consumption with [^18^F]FAZA after IACS-010759 (-80%) [35], changes in redox with [^18^F]FSPG changes after Doxil treatment (-42%) [36] or with FDG response after chemotherapy (-38%) [37]. In addition, we were able to detect the response earlier than previous studies, with NIS measuring changes within approximately 90 min post-treatment, compared to 4 h for [^18^F]fluoroBnTP PET [34] and 24 h for FDG PET [37], [^18^F]FSPG [36], and [^18^F]FAZA [35]. These results suggest that [^18^F]TFB PET measurements of NIS activity may be a more sensitive detection tool than standard radionuclide imaging approaches measuring metabolic response.

The limitation of using NIS-mediated radioactivity uptake as a metabolic response biomarker is that NIS is expressed only in a subset of non-malignant mammalian tissue types, including stomach epithelium, thyroid and lactating mammary glands [9, 13], which necessitates the modification of the investigated tumour models to express NIS. Therefore, the usefulness of NIS is principally as a tool for preclinical in vivo assessment and not as a clinical biomarker. However, some subtypes of cancer cells, such as breast, urological, and thyroid cancers also upregulate NIS as part of tumorigenesis, so it is conceivable that a similar approach could be applied clinically using PET imaging to image endogenously expressed human NIS isoform [38]. In these cases, this endogenous expression could be used in a similar way to determine early metabolic response to drug targeting metabolic stress. Thyroid scintigraphy using Tc-99m pertechnetate or iodide-123 is frequently used to image NIS function in patients providing a facile route for translation.

Further research is warranted to gain a deeper understanding of the impact of NIS expression on cell phenotype and drug response. One potential concern is that NIS expression could directly sensitize cancer cells to treatment, which might lead to false positive results when measuring the response to novel treatments. If NIS expression alone enhances the sensitivity of cancer cells to certain drugs, it could confound the interpretation of NIS activity as a biomarker solely for metabolic stress induced by the treatment. Additionally, the influence of changes in plasma membrane potential on NIS activity requires further investigation. As NIS activity is also mediated by the electrical potential across the plasma membrane [39], understanding how variations in this potential affect NIS function is crucial for accurately interpreting NIS activity measurements and distinguishing them from changes solely caused by metabolic stress. Despite these challenges, our studies have demonstrated the capability to differentiate between tumours with similar NIS expression but varying sensitivity to oxidative phosphorylation inhibition. This finding suggests the potential value of NIS as a pharmacodynamic biomarker in specific contexts.

## Conclusions

We have demonstrated here that the imaging reporter gene NIS provides a rapid and sensitive readout of ATP re-synthesis disruption that can be imaged in vivo using PET and propose the novel use of NIS as a metabolic sensor for drugs that lead to ATP depletion. NIS could facilitate in vivo testing of metabolic treatments targeting energetic pathways, essential for determining metabolic drug potency and expediting metabolic drug development.

### Supplementary Information

**Additional file 1: Supplementary Figure 1.** Illustrative images showing H&E staining of tumour tissue fixed shortly after the completion of vehicle or IACS-10759 treatment, as stated on the label in the left-hand corner of the image (Vehiclex1, IACSx1: fixed 2 hours from the administration of a single dose; Vehiclex6, IACSx6: fixed 24 hours from the administration of the last one of six, once-daily doses). **Supplementary Figure 2.** [^18^F]TFB PET imaging of sodium iodide symporter (NIS) expressing sensitive (A549-LN; *n* = 4) and resistant (H358-LN; *n* = 3) tumours at baseline and 24 hours later, immediately after oral administration of a single dose of 20mg/kg IACS-10759. Two-way, repeated measures ANOVA with Šídák's multiple comparisons analysis was performed to test statistical significance of the results. P value classifications are summarized as follows: **, P∈(0.001–0.01 〉. Each data point represents SUVmax calculated for a single tumour/animal and time-point.

## Data Availability

All data generated or analysed during this study are included in this published article. The datasets used and/or analysed during the current study are available from the corresponding author on reasonable request.

## Abbreviations

2-DG: 2-Deoxyglucose
ATP: Adenosine triphosphate
ADP: Adenosine diphosphate
AMP: Adenosine monophosphate
DMSO: Dimethyl sulfoxide
fLuc: Firefly luciferase
NIS: Sodium iodide symporter
HSV1-tk: Herpes simplex virus type 1 thymidine kinase
PET: Positron emission topography
TFB: [^18^F]tetrafluoroborate
