# Supplementary material for: Positron emission tomography imaging of the sodium iodide symporter senses real-time energy stress in vivo
Source: Cancer Metab. 2023 Sep 7;11:14. doi: 10.1186/s40170-023-00314-2 (PMC10486058; doi:10.1186/s40170-023-00314-2)
Supplement: Supplementary file 1 — Additional file 1: Supplementary Figure 1. Illustrative images showing H&E staining of tumour tissue fixed shortly after the completion of vehicle or IACS-10759 treatment, as stated on the label in the left-hand corner of the image (Vehiclex1, IACSx1: fixed 2 hours from the administration of a single dose; Vehiclex6, IACSx6: fixed 24 hours from the administration of the last one of six, once-daily doses). Supplementary Figure 2. [18F]TFB PET imaging of sodium iodide symporter (NIS) expressing sensitive (A549-LN; n = 4) and resistant (H358-LN; n = 3) tumours at baseline and 24 hours later, immediately after oral administration of a single dose of 20mg/kg IACS-10759. Two-way, repeated measures ANOVA with Šídák's multiple comparisons analysis was performed to test statistical significance of the results. P value classifications are summarized as follows: **, P∈(0.001–0.01 〉. Each data point represents SUVmax calculated for a single tumour/animal and time-point. [file 40170_2023_314_MOESM1_ESM.pdf]

Supplementary Figure 1

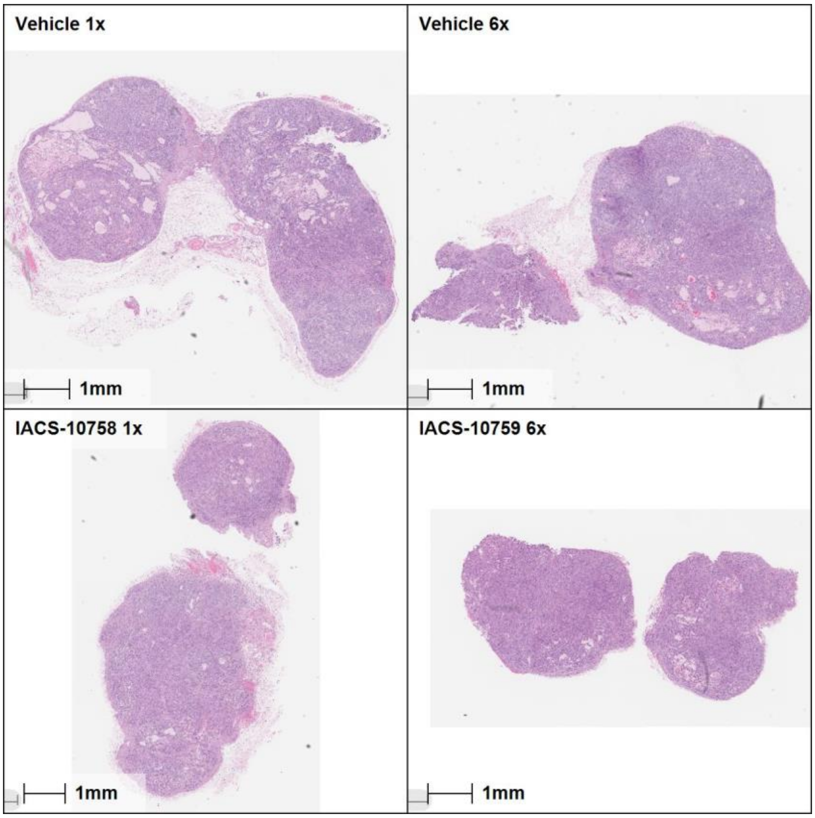

Supplementary Figure 1.

Illustrative images showing H&E staining of tumour tissue fixed shortly after the completion of vehicle or IACS-10759 treatment, as stated on the label in the left-hand corner of the image (Vehiclex1, IACSx1: fixed 2 hours from the administration of a single dose; Vehiclex6, IACSx6: fixed 24 hours from the administration of the last one of six, once-daily doses).

Supplementary Figure 2

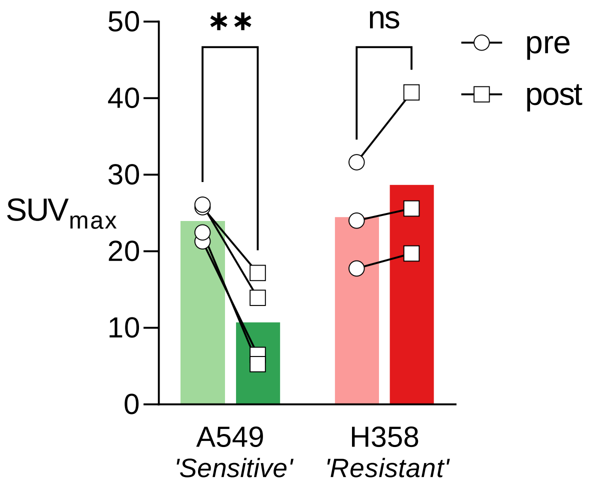

Supplementary Figure 2.

[^{18}F]TFB PET imaging of sodium iodide symporter (NIS) expressing sensitive (A549-LN; n = 4) and resistant (H358-LN; n = 3) tumours at baseline and 24 hours later, immediately after oral administration of a single dose of 20mg/kg IACS-10759. Two-way, repeated measures ANOVA with Šídák's multiple comparisons analysis was performed to test statistical significance of the results. P value classifications are summarized as follows: **, P∈(0.001–0.01 ⟩. Each data point represents SUV_{max} calculated for a single tumour/animal and time-point.
